# Possible Involvement of *µ* Opioid Receptor in the Antidepressant-Like Effect of Shuyu Formula in Restraint Stress-Induced Depression-Like Rats

**DOI:** 10.1155/2015/452412

**Published:** 2015-03-02

**Authors:** Fu-rong Wang, Ming-qi Qiao, Ling Xue, Sheng Wei

**Affiliations:** ^1^Department of Pharmacology, College of Basic Medicine, Shandong University of Traditional Chinese Medicine, Jinan 250355, China; ^2^Key Laboratory for Classical Theory of Traditional Chinese Medicine of Education Ministry, Shandong University of Traditional Chinese Medicine, Jinan 250355, China

## Abstract

Recently *μ* opioid receptor (MOR) has been shown to be closely associated with depression. Here we investigated the action of Shuyu, a Chinese herbal prescription, on repeated restraint stress induced depression-like rats, with specific attention to the role of MOR and the related signal cascade. Our results showed that repeated restraint stress caused significant depressive-like behaviors, as evidenced by reduced body weight gain, prolonged duration of immobility in forced swimming test, and decreased number of square-crossings and rearings in open field test. The stress-induced depression-like behaviors were relieved by Shuyu, which was accompanied by decreased expression of MOR in hippocampus. Furthermore, Shuyu upregulated BDNF protein expression, restored the activity of CREB, and stimulated MEK and ERK phosphorylation in hippocampus of stressed rats. More importantly, MOR is involved in the effects of Shuyu on these depression-related signals, as they can be strengthened by MOR antagonist CTAP. Collectively, these data indicated that the antidepressant-like properties of Shuyu are associated with MOR and the corresponding CREB, BDNF, MEK, and ERK signal pathway. Our study supports clinical use of Shuyu as an effective treatment of depression and also suggests that MOR might be a target for treatment of depression and developing novel antidepressants.

## 1. Introduction

Nowadays, depression and anxiety afflict 350 million people worldwide. The chronic, recurrent, and life-threatening (due to the risk for suicide) nature of depression contributes substantially to the global burden of disease and disability, expected to even worsen in the next decades [1]. Currently, several promising hypotheses of depression and antidepressant action are largely based on dysfunction of the hypothalamic-pituitary-adrenal axis, aberrant of monoamine neurotransmitter release, disorders of glutamic acid circulation, and so on. However, selective serotonin reuptake inhibitors (SSRIs), the most popular antidepressants nowadays, are effective in only 40–50% patients [2]. Therefore, to investigate the underlined mechanisms of depression and related drugs is crucial.

*μ* opioid receptor (MOR), one of the four kinds of known opioid receptors, is well known to play vital roles in a plethora of physiology and pathology processes, such as pain, tolerance, drug addiction, immunity, and respiratory depression. Recently, plentiful researches have discovered the important functions of MOR in emotional regulation, etiology, and treatment of some mood disorders. In patients with major depressive disorder, both neutral state and sustained sadness condition were associated with a statistically significant MOR activation [3]. Of note, both stressful life events on symptoms of major depression [4] and rates of response to antidepressants and consequent remission from major depressive disorder [5] are influenced by variation in the MOR gene. Specifically, knock-out of MOR in the mice abolished the antidepressant effects of venlafaxine [6]. In addition, opioid compounds decreased the immobility of the mice and increased their curling behavior in tail suspension test, while antidepressants that inhibit noradrenaline and/or serotonin reuptake only decreased the immobility of mice but showed no effect on their curling behavior [7]. Gassaway et al. even considered the atypical antidepressant and neurorestorative agent tianeptine was a MOR agonist [8]. These results indicate that MOR could be a key mediator in the pathology of depression and in antidepressant-like effects.

In recent years, traditional Chinese medicine has become one kind of much-used therapy for mild-to-moderate depression. Plenty of herbs and herbal preparations have been widely studied and used as an effective alternative in the treatment of depression and other neuropsychiatric disorders, mainly due to the elevated efficacy, reduced incidence of adverse effects, and improved outcomes [9]. Shuyu, a Chinese herb formula prescription, is composed of four herbs,* Paeonia suffruticosa* (Mu Dan Pi),* Bupleurum chinensis* (Chai Hu),* Cyperus rotundus* (Xiang Fu), and* Glycyrrhiza uralensis Fisch* (Gan Cao). Shuyu has been clinically used for the treatment of mental disorders for long time. Scientific pharmacological studies have revealed that some components, such as* Paeonia suffruticosa*,* Bupleurum chinensis*,* Cyperus rotundus*, and* Glycyrrhiza uralensis Fisch,* have antidepressant effects [10–13]. Our previous study demonstrated that Shuyu possesses an antidepressant-like action by normalizing the expression of 5-HT3B receptor in hippocampus of chronic mild stress-induced depressive-like animals [14]. However, whether MOR and the related signal pathway are involved in the antidepressant-like role of Shuyu remains unclear.

Restraint stress, including acute and chronic stress, is frequently employed to induce depressive-like behavioral states in rodents [15, 16]. In this study we aimed to examine the effect of Shuyu on repeated restraint stress-induced depression-like animals. More importantly, we intended to investigate whether the antidepressant effect of Shuyu is mediated by MOR, and the depression-associated signal molecules, that is, CREB (cAMP response element binding protein), BDNF (brain derived neurotrophic factor), MEK, and ERK.

## 2. Materials and Methods

### 2.1. Animals

Eight-week-old female Sprague-Dawley rats (220–250 g) were purchased from Animal Center, Shandong University of Traditional Chinese Medicine (Shandong Province, China). Animals were housed under conditions of constant temperature (23 ± 2°C) and humidity (55 ± 10%), with food and water available* ad libitum* and with a normal 12 h/12 h light/dark schedule with the lights on between 07:00 am and 07:00 pm. The animals were allowed one week to acclimatize themselves to the housing conditions before the beginning of experiments. All animal procedures were performed in accordance with the guidelines for the Care and Use of Laboratory Animals published by the US National Institutes of Health (NIH) and were approved by the Animal Care Committee of Shandong University of Traditional Chinese Medicine.

### 2.2. Plant Materials and HPLC Analysis

A herbarium voucher sample that consists of Shuyu was manufactured by Qingdao Haichuan Innovative Bionatural Medicine Research Center (Shandong, China). The herbs that consist of Shuyu are* Paeonia suffruticosa* (Mu Dan Pi, root, from Shandong) 1.33 g,* Bupleurum chinensis* (Chai Hu, root, from Henan) 1.33 g,* Cyperus rotundus* (Xiang Fu, root, from Sichuan) 1.11 g, and* Glycyrrhiza uralensis Fisch* (Gan Cao, root, from Gansu) 1.11 g. All of the herbs were carefully identified by Professor Huiyun Zhang of Shandong University of TCM (Jinan, China). The four components were dried and mixed in proportion and decocted in hot distilled water twice for 2 h. The filtrates were combined and condensed by spray-drying and low-temperature vacuuming and made into drug powder eventually (yield: 75.28%). This procedure followed the TCM canon and remained consistent with the clinical preparation method.

**Figure 1:**
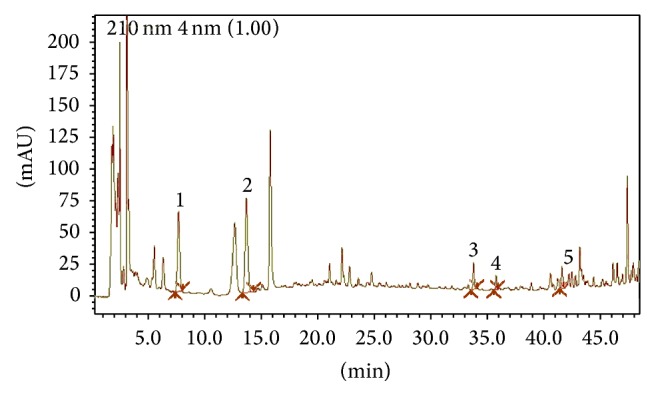
HPLC chromatogram of Shuyu preparation. Six major constituents were detected. The concentration, linearity, limit of detection (LOD), limit of quantification (LOQ), and interbatch variation (CV) are listed in Table 1.

To ensure the quality and stability of the SNK solution, we used high performance liquid chromatography (HPLC) to identify the active compounds. The HPLC analysis was performed by Prominence LC-20AD detector and Waters symmetry C18 column (250 mm × 4.6 mm, ID 5 *μ*m) with the column temperature at 30°C. The mobile phase was acetonitrile −0.1% phosphate water in gradient mode, with the flow rate of 1.2 mL/min. All solvents were filtered through a 0.45 *μ*m filter (Millipore, Bedford, MA, USA) before use. The effluent was monitored at 210 nm by a UV detector. The HPLC chromatogram of Shuyu preparation and concentrations of the major constituents are shown in Figure 1 and Table 1.

### 2.3. Groups and Drug Administration

Shuyu was dissolved with distilled water and administered intragastrically. D-Phe-Cys-Tyr-D-Trp-Arg-Thr-Pen-Thr-NH2 (CTAP, Sigma-Aldrich, St. Louis, MO, USA), a highly selective MOR antagonist, was dissolved in sterile dH2O at a concentration of 5 *μ*g/*μ*L and stored at 4°C in a dark place.

All the stressed rats were randomly divided into four groups with 14 animals for each: stress-induced depression-like model group and model group treated either with Shuyu and/or CTAP. Another 28 rats were left as normal controls or CTAP treated normal animals, respectively.

Shuyu (0.4 g/kg BW) was gavaged to the rats once daily 1 hour before restraint stress for 5 days. The dose was chosen based on our previous study in which Shuyu administered at a dose of 0.4 g/kg exerted antidepressant-like effect [14]. Other rats were orally administered dH2O of the same volume.

CTAP was intracerebroventricularly injected (2 *μ*g/kg BW) to rats for 5 consecutive days just before the sacrifice. Other rats underwent the same surgical procedures, but same volume of saline was injected instead of CTAP.

### 2.4. Repeated Restraint Stress (RRS)

Depression-like behavioral phenotypes can be induced in animal models treated with repeated stress, such as with 45 min × 7 d [17], 2 h × 10 d [18], or 2 h × 14 d restraint [19], and others. Repeated restraints have a superior feasibility in handling experimental procedures compared to other models, although the concept of effective doses of stress threshold and optimized stress strength has not been established. Thus in this study the reproducible animal model of stress-induced depression-like behaviors was created by administrating a 5-day restraint stress (4 h/day) to rats and subsequently performing behavioral assessments.

The restraint stress procedure was carried out for 4 h per day from 10:00 am to 14:00 pm for 5 consecutive days. Rats were placed individually in a transparent plastic cylinder with a diameter of 3 cm and height of 12 cm. This restrained all physical movement but allowed for ample air. The animals were deprived of food and water during the entire period of exposure to stress. The following parameters were measured to monitor the effects of the stress 60 min after the last restraint: changes of body weight from the beginning step of restraint stress to sacrifice of animals, forced swimming test, and open field test. Normal control and CTAP treated normal rats were not restrained and handled during tests.

### 2.5. Forced Swimming Test (FST)

The forced swimming test was conducted to all groups. Briefly, individual rat was forced to swim in an open cylinder (25 cm in height, 10 cm in diameter) with water filled to 19 cm at 24~26°C. The total duration of immobility for the last 4 min was recorded by a single observer for a single 6 min test session. Rats were considered immobile when they made no attempts to escape (in seconds), except for the movements necessary to keep their heads above the water. A decrease in the duration of immobility is indicative of an antidepressant-like effect and an increase of immobility time, when compared to the control group, is considered a depressive-like effect.

### 2.6. Open Field Test (OFT)

To access the possible effects of Shuyu on locomotor activity, rats were evaluated in the OFT paradigm. The open field apparatus was a wooden arena (40 × 60 × 50 cm) and divided into a central field (center, 20 × 20 cm) and a border field (periphery). Animals were individually placed in the central field, permitted free exploration, and monitored by a video tracking system. The number of squares crossed with all paws (crossing, horizontal locomotor activity) and times of standing on hind limbs (rearing, vertical locomotor activity) were counted in a 6 min session. The apparatus was cleaned by a detergent and dried prior to occupancy by next rat. The recorded videos were scored by two observers and the average value was taken.

### 2.7. Intracerebroventricular (*i.c.v.*) Cannula Implantation

The rats were anesthetized with chloral hydrates (50 mg/kg,* i.p.*) and their heads were fixed in a stereotaxic frame. The skull was exposed and two small holes were drilled and injection cannula was lowered. For microinjection into the lateral ventricle, the coordinates were ±1.6 mm from the midline, 0.9 mm posterior to bregma, and 2.0 mm ventral to dura mater, with injector aimed 4.0 mm ventral to the dura. Injection cannula was connected to a Hamilton syringe attached to a microinjector unit. Animals were habituated to dummy cannula removal and given 7 days of recovery and handling before the start of behavioral training.

### 2.8. Immunofluorescence Staining

Some animals were terminally anesthetized with pentobarbital sodium (80 mg/kg,* i.p.*) and perfused through the ascending aorta with saline, followed by freshly prepared 4% paraformaldehyde in a 0.1 M PBS (pH 7.4, 4°C). After perfusion, the hippocampus was isolated immediately and postfixed in the same fixative overnight. All of the hippocampi were embedded in paraffin and cut serially into 5 *μ*m sections. After antigen retrieval in citrate buffer, the sections were blocked with 10% goat serum/PBS for 1 h at room temperature and incubated overnight at 4°C with rabbit monoclonal anti-rat MOR (1 : 500, Abcam, Cambridge, UK). Then the sections were incubated with the secondary antibody, FITC-conjugated AffiniPure donkey anti-mouse IgG (Jackson ImmunoResearch Laboratories, Bar Harbor, ME, USA). Samples were observed and digital images were obtained with a fluorescence microscope (Leica DMIRE2 Microsystems GmbH, Wetzlar, Germany). A negative control was obtained by incubating with PBS instead of primary antibody in each experiment.

Quantitative measurement was done by two researchers blind to experimental groups, and the average data were obtained. At 400x, the numbers of MOR-like immunopositive neurons in hippocampus were counted within a calibrator (50 *μ*m × 50 *μ*m) in five sections from each rat. The criterion for acceptance as a neuron was the clear differentiation from background staining and a profile of soma.

### 2.9. Western Blot Analysis

Other animals were anesthetized and hippocampi were removed quickly for western blot analysis. The isolated hippocampus was homogenized in ice-cold lysis buffer containing a mixture of protease inhibitors and phosphatase inhibitors (Sigma-Aldrich, St. Louis, MO, USA). The protein concentrations of the lysate were determined using a BCA Protein Assay kit (Pierce, Rockford, IL, USA) following the manufacturer's protocol.

Protein samples (30 *μ*g) were loaded for each lane and separated on a SDS-PAGE gel (10% gradient gel; Bio-Rad, Hercules, CA, USA) and transferred onto nitrocellulose membranes (Amersham Pharmacia Biotech, Little Chalfont, UK) at a constant power of 100 V for 2 h at 4°C. The filters were subsequently blocked with 5% nonfat dry milk and then incubated overnight at 4°C with a primary antibody, rabbit monoclonal anti-rat MOR (1 : 1000, Abcam, Cambridge, UK). After washing, the membrane was incubated with a HRP-conjugated secondary antibody (1 : 2000; Jackson ImmunoResearch, PA, USA) for 1 h at room temperature. The blots were visualized in an ECL solution (Amersham Pharmacia Biotech, Piscataway, NJ, USA) for 2 min. Finally, the membrane was reblotted with mouse anti-*β*-actin monoclonal antibody (Abcam, Cambridge, UK). The intensity of immunoblot bands was detected by densitometry with Alphalmager TM 2000. Similar procedures were accomplished for detection of ERK1/2, P-ERK1/2, CREB, P-CREB, and BDNF protein expression.

### 2.10. Statistical Analyses

All data were expressed as mean ± SEM. Differences among groups were analyzed by one-way ANOVA, followed by post-hoc Bonferroni's or Dunnett's test. *P* < 0.05 was considered statistically significant. The Statistical analyses were performed with SPSS 17.0 for windows (SPSS Inc., Chicago, IL, USA).

## 3. Results

### 3.1. Shuyu Relieves the Behavioral Changes of RRS Animals

**Figure 2:**
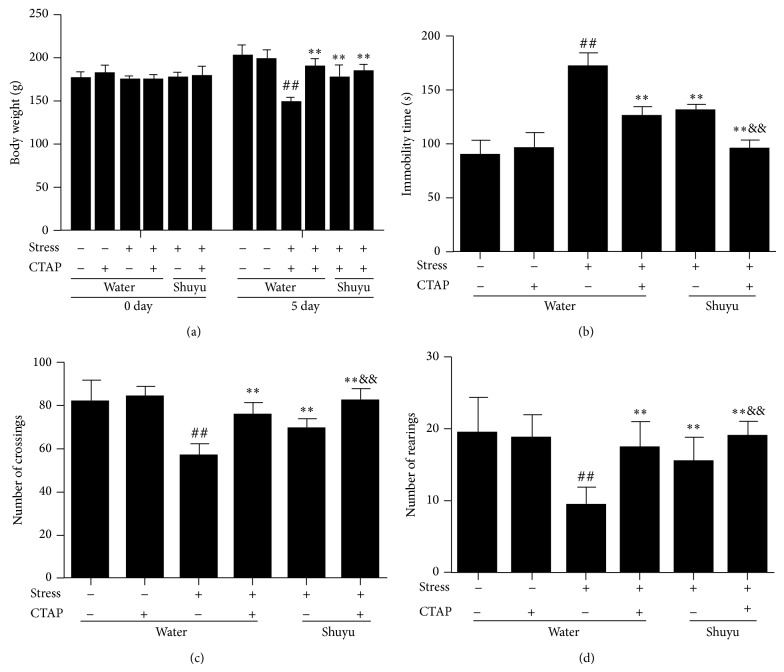
Behavior changes of stressed rats treated with/without Shuyu and/or CTAP. Body weight was monitored before stress (0 day) and just before sacrifice (5 day) (a). Forced swimming test and open field test were performed just before sacrifice. Duration of immobility (b), number of crossings (c), reflecting range of motion, and the number of rearings (d), reflecting exploratory behaviors were recorded as explained in the Materials and Methods. Values are shown as mean ± SEM (each *n* = 14). ^##^ *P* < 0.01 compared with the normal control group. ^**^ *P* < 0.01 compared with RRS group. ^&&^ *P* < 0.01 compared with Shuyu treated RRS group.

Three biological parameters were measured to monitor the effectiveness of the RRS protocol: body weight gain, forced swimming test, and open field test. There is no obvious difference of baseline body weight among rats from different groups, while after five days body weight in the rats of RRS model decreased significantly compared with normal rats. However, at the end of experiment, body weight of Shuyu and/or CTAP treated rats was obviously higher than that of the RRS rats (Figure 2(a)). That is to say, exposure to RRS decreased the body weight gain, which could be recovered by Shuyu administration.

We next performed FST to evaluate the depressive-like behavior and investigate the antidepressive effects of Shuyu. As shown in Figure 2(b), exposure to RRS significantly prolonged the duration of immobility in comparison with normal control rats (*P* < 0.01). After Shuyu and/or CTAP treatment, the immobility time decreased markedly, as compared to RRS-induced depressive rats (all *P* < 0.01).

In the open field test, the baseline locomotor activities were similar among all the rats (data not shown). After stress exposure, the RRS rats showed a significant decrease in the number of square-crossings and rearings versus the normal rats (*P* < 0.01). Treatment with either Shuyu or CTAP obviously restored the number of square-crossings and rearings of the RRS rats (both *P* < 0.01) (Figures 2(c) and 2(d)). Moreover, the depressive-related behaviors were further ameliorated by the combination use of Shuyu and CTAP.

These indicate that rats subjected to repeated restraint stress for five days exhibited a significant depression phenotype, while Shuyu may relieve the behavioral changes of the RRS animals.

### 3.2. Shuyu Decreases the Protein Expression of MOR in Hippocampus of RRS Rats

**Figure 3:**
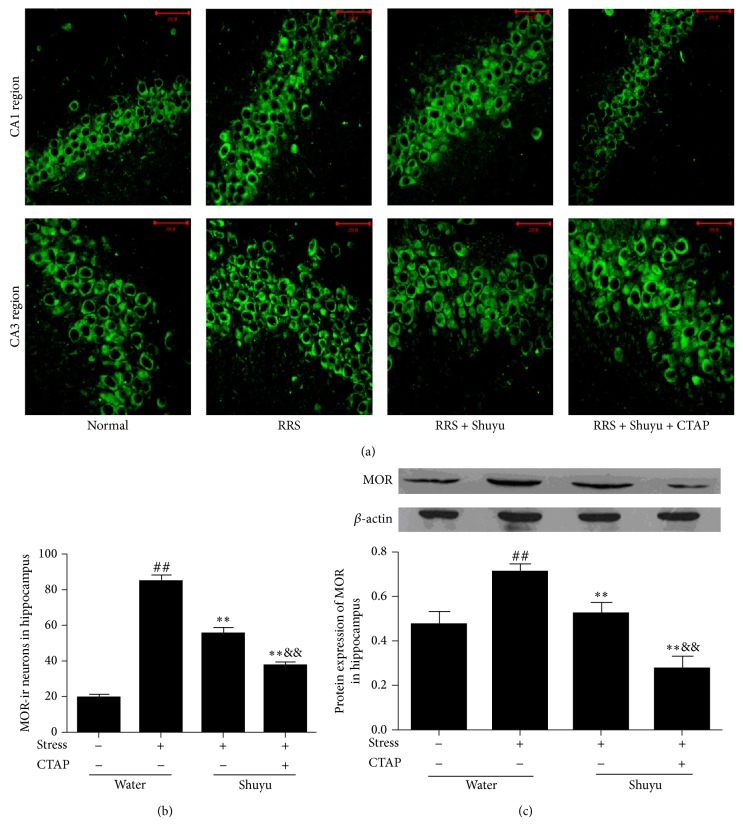
Expression of MOR in the CA1 and CA3 region of hippocampus detected by immunofluorescence staining (a) (×400) and by Western blot analysis (c). The numbers of MOR-immunoreactive neurons in the hippocampus were calculated (b). The intensity of immunoblot bands were quantified by Alphalmager TM 2000 and presented as mean ± SEM (each *n* = 14). ^##^ *P* < 0.01 compared with the normal control group. ^**^ *P* < 0.01 compared with RRS group. ^&&^ *P* < 0.01 compared with Shuyu treated RRS group.

Figure 3(a) showed the photomicrographs of MOR expression in the hippocampal CA1 and CA3 region. In normal rats, pyramidal cells in the hippocampal CA1 and CA3 region are tightly packed and regularly shaped, and the MOR positive cells are relatively few. But in the RRS rats, pyramidal cells in the same region are disorderly arranged, some even shrank, while the number of MOR positive cells increased significantly. After Shuyu treatment, the morphological abnormalities of pyramidal cells were restored, while the number of MOR positive cells decreased, and further reduced by addition of CTAP (Figure 3(b)).

We further examined the effect of Shuyu on MOR protein expression in hippocampus of RRS rats with Western blot analysis. Significant elevation of MOR expression was found in the RRS rats compared with normal control animals (*P* < 0.01). Administration with Shuyu effectively counteracted the effect of RRS and reduced the expression of hippocampal MOR (*P* < 0.01). CTAP injection significantly enhanced this reduction (*P* < 0.01) (Figure 3(c)).

### 3.3. Shuyu Upregulates BDNF Protein Expression in Hippocampus of RRS Rats

**Figure 4:**
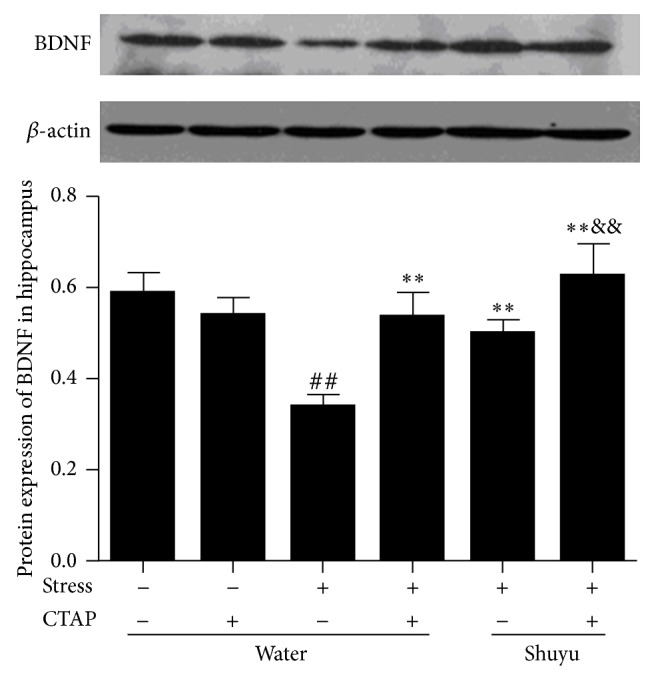
BDNF protein expression in the rat hippocampus examined by Western blot analysis. The intensity of immunoblot bands was quantified by Alphalmager TM 2000 and presented as mean ± SEM (each *n* = 14). ^##^ *P* < 0.01 compared with the normal control group. ^**^ *P* < 0.01 compared with RRS group. ^&&^ *P* < 0.01 compared with Shuyu treated RRS group.

BDNF, the most abundant neurotrophins, plays an important role in neuronal genesis and plasticity of vertebrate brain. Series of clinical and basic studies have demonstrated that depression decreases BDNF expression which can be reversed or blocked by antidepressant treatment. Recently more and more attention has been put on the BDNF and MOR and depression. Here we examined the protein expression of BDNF in hippocampus using Western blot analysis. From Figure 4 we can see that the RRS animals showed significantly less expression of BDNF protein than normal rats (*P* < 0.01), while both Shuyu and CTAP reversed the reduction to almost the baseline level. CTAP itself did not affect BDNF protein expression in nonstressed rats but strengthened the effect of Shuyu. This may indicate that the upregulation of BDNF protein expression in RRS rats by Shuyu is related with MOR.

### 3.4. Shuyu Restores the Activity of CREB in Hippocampus of RRS Rats

**Figure 5:**
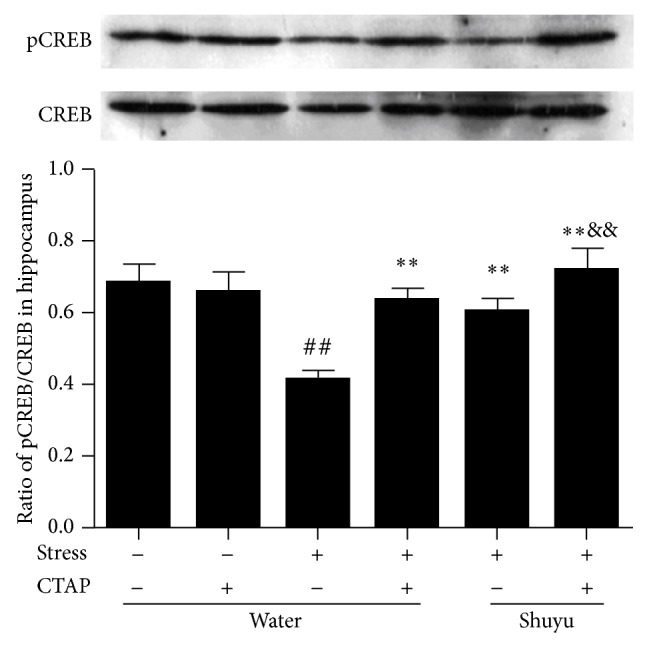
Phosphorylation of CREB in the rat hippocampus presented by the ratio of pCREB/CREB protein expression as examined using Western blot analysis. The intensity of immunoblot bands was quantified by Alphalmager TM 2000 and expressed as mean ± SEM (each *n* = 14). ^##^ *P* < 0.01 compared with the normal control group. ^**^ *P* < 0.01 compared with RRS group. ^&&^ *P* < 0.01 compared with Shuyu treated RRS group.

As we know, the promoter region of BDNF exon IV contains specific binding sites for the transcriptional regulators CREB. Upon phosphorylation, phosphorylated CREB (pCREB) becomes associated with CRE elements in the exon IV promoter of BDNF, thus inducing the transcription of the BDNF transcript. Therefore, we measured the level of phospho-CREB, the active form of the protein, and CREB expression in hippocampus using Western blotting. The ratio of pCREB/CREB represents the phosphorylation status of CREB, that is, the activity of CREB.  Figure 5 shows that RRS led to a significant reduce of the ratio of pCREB/CREB (*P* < 0.01), which was normalized by Shuyu administration or CTAP injection (both *P* < 0.01). Furthermore, addition of CTAP elevated the pCREB/CREB ratio more obviously than Shuyu alone. This suggests that Shuyu may restore the activity of CREB in the hippocampus of RRS rats, which was tightly associated with the MOR.

### 3.5. Shuyu Stimulates MEK Phosphorylation in Hippocampus of RRS Rats

**Figure 6:**
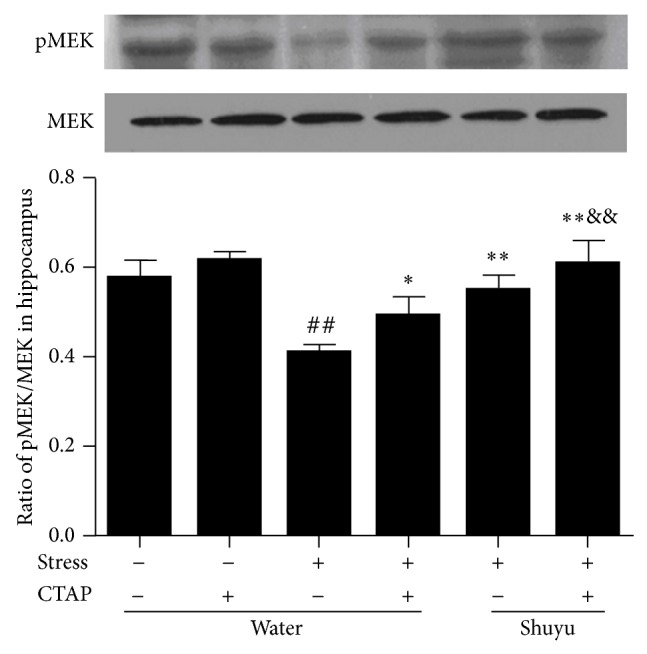
Phosphorylation of MEK in the rat hippocampus presented by the ratio of pMEK/MEK protein expression as examined using Western blot analysis. The intensity of immunoblot bands was quantified by Alphalmager TM 2000 and expressed as mean ± SEM (each *n* = 14). ^##^ *P* < 0.01 compared with the normal control group. ^*^ *P* < 0.05, ^**^ *P* < 0.01 compared with RRS group. ^&&^ *P* < 0.01 compared with Shuyu treated RRS group.

We further conducted Western blot analysis to investigate the effect of Shuyu on the protein expression of pMEK and MEK, a crucial dual-specificity kinase underneath the BDNF signal pathway. As can be seen in Figure 6, pMEK protein expression was significantly downregulated in the hypothalamus of water-administered stressed rats compared with that of water-administered nonstressed rats (*P* < 0.01). Shuyu treatment significantly ameliorated the stress-induced decrease of pMEK expression (*P* < 0.01). CTAP injection further elevated the hippocampus pMEK expression in Shuyu treated RRS rats (*P* < 0.01). This indicates that MOR involved the elevation of pMEK protein expression, namely, the stimulation of MEK phosphorylation induced by Shuyu in RRS rats.

### 3.6. Shuyu Induces ERK1/2 Phosphorylation in Hippocampus of RRS Rats

**Figure 7:**
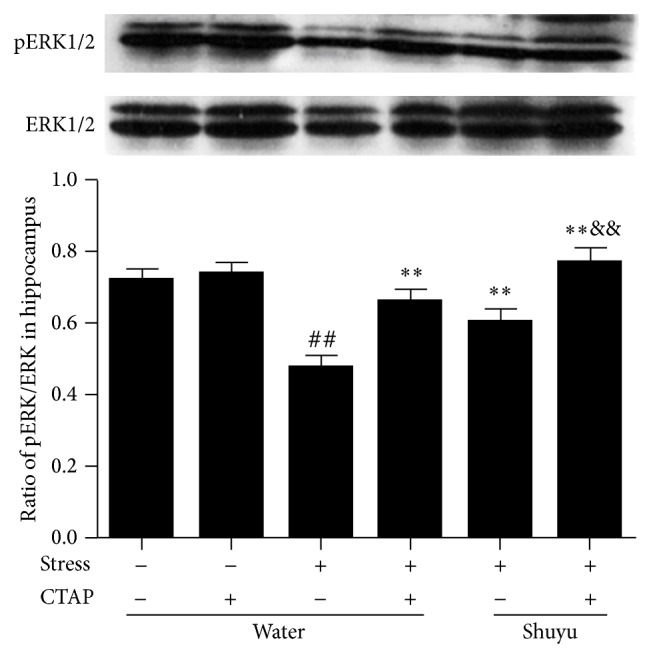
Phosphorylation of ERK1/2 in the rat hippocampus presented by the ratio of pERK1/2 and ERK1/2 protein expression as examined using Western blot analysis. The intensity of immunoblot bands was quantified by Alphalmager TM 2000 and expressed as mean ± SEM (each *n* = 14). ^##^ *P* < 0.01 compared with the normal control group. ^**^ *P* < 0.01 compared with RRS group. ^&&^ *P* < 0.01 compared with Shuyu treated RRS group.

It is well known that MEK is the immediate upstream regulator of ERK1/2. By phosphorylating at Ser and Thr residues, MEK activates ERK1/2, which then phosphorylates cytoplasmic and nuclear substrates, such as CREB. Thus we examined the phosphorylation status of ERK1/2 by detecting the ratio of pERK/ERK with Western blot analysis. As can be seen from Figure 7, RRS-induced depression-like model rats were associated with a significant decrease of pERK/ERK when compared with normal control rats. The RRS-induced decrease of pERK/ERK was significantly elevated in both Shuyu and CTAP treated rats, and to a greater extent with the combined use. This implies that the increased phosphorylation of ERK1/2 in RRS animals caused by Shuyu is concerned with MOR.

## 4. Discussion

In the present study we found that Shuyu, a traditional Chinese prescription, prevented depressive-like behaviors in restraint stress stimulated rats. More importantly, this effect is tightly correlated with *μ* opioid receptor and the consecutive signal cascades, CREB, BDNF, MEK, and ERK. Although MOR has been considered to be involved in the development of depression, none of the related practical strategies has been proposed in clinics. Therefore, our study first provided evidences for the herbal treatment of depression with MOR as the potential target.

As stressful life events have been reported to facilitate the evolution of depression disorders, acute or chronic restraint stress in rodents has been widely used as a model of depression [15, 16]. Using restraint stress as a stress-induced model of depression possesses several major advantages. First, restraint stress represents the most severe type of stress which combines both emotional and physical components. Second, restraint stress produces an inescapable physical and mental stress which seldom accompanies adaption. In the present study, repeated restraint stress caused depressive-like behavior, as evidenced by body weight gain, FST, and OFT. Moreover, compelling evidences have revealed sex differences in anxiety and depressive disorders, with females more than twice as likely to be afflicted [20]. Thus it is crucial to clarify that our study was performed in female rats.

Recent studies have indicated the crucial roles of MOR in the occurrence, progression, and severity of depression, and the significant actions of medications on the expression and function of MOR. Acute pharmacological activation of the MOR reduced depressive-like behaviors in mice, such as the immobility time in forced swimming test [21], immobility, swinging, and curling behavior in tail suspension test [7]. In this study we found elevated expression of MOR in the hippocampus of RRS-exposed rats with both immunofluorescence and Western blot analysis. Our results are in line with studies showing decreased depressive-like behaviors in the forced swimming test [22], lessened immobility in a 15 min tail suspension test [23], and reduced aversion to social contact [24] in MOR knock-out mice. Besides, Gonzales et al. reported that immediately following 30 minutes of acute restraint stress, male rats had higher levels of phosphorylated MOR immunoreactivity within the dentate gyrus region [25]. Also, Yamamota et al. showed increased MOR mRNA levels in the rat hypothalamus and midbrain after 4 h restraint stress for 2 days [26]. Similar results come from clinical researches. MOR expression was increased in frontal and temporal cortex and caudate nuclei in suicide victims diagnosed with depression compared with controls who died of sudden death with no history of psychiatric disorders [27]. This suggests that the MOR-mediated mechanisms of depression may be more complex than previously anticipated. Possibilities of the discrepancy may include differences of models and brain regions, duration of treatment or negative emotional states, and mood state of the subjects or animals, and so on.

Shuyu, a Chinese herbal medicine composed of four herbs (*Paeonia suffruticosa*,* Bupleurum chinensis*,* Cyperus rotundus*, and* Glycyrrhiza uralensis Fisch*), is clinically applied in the treatment of depression-like symptoms and other mental disorders. Based on the traditional Chinese medicine theories, Shuyu exhibits therapeutic effects on depression mainly by dispersing stagnated liver qi and relieving qi stagnation. Recently, modern scientific studies have revealed the antidepressant effects of* Paeonia suffruticosa*,* Bupleurum chinensis*,* Cyperus rotundus*, and* Glycyrrhiza uralensis Fisch*, and formula with these herbs as the main constituents. Extracts from* Bupleurum chinensis* or* Cyperus rotundus*,* Paeonia suffruticosa* and its total glycosides, and* Glycyrrhiza uralensis Fisch* and its derivation liquiritin have shown the abilities of preventing depression-like behaviors in animals exposed to repeated restraint stress and chronic unpredictable mild stress [11, 12], and in tail suspension test and forced swimming test [10, 13]. These data confirmed our results of Shuyu's effects on RRS-induced depression-like animals. However, the antidepressant mechanisms of these natural products are always considered via inhibition of monoamine oxidase activity, modulation of the function of hypothalamic-pituitary-adrenal axis, and upregulation of neurotrophins and neurotransmitters, and so on. In this study we first revealed that the combined use of* Paeonia suffruticosa*,* Bupleurum chinensis*,* Cyperus rotundus*, and* Glycyrrhiza uralensis Fisch* in the treatment of depression-like activities may be mediated by inhibition of MOR. Similarly, chronic antidepressant treatment causes reduction of MOR protein expression in the guinea pig hippocampus [28] and MOR binding and functional coupling to G proteins in the amygdale of fawn-hooded rats [29]. These results together with ours indicate that antidepressants may regulate opioid systems and that opioid systems may be important to the antidepressant effects of some drugs.

It is well known that BDNF mediated signaling pathways are implicated in the neuroplasticity alterations evoked by depression and antidepressants. Both serum BDNF levels in depressed subjects [30] and hippocampal BDNF expression in stress stimulated mice [24] were lower than in normal controls and increased with antidepressants treatment. Furthermore, impaired neuronal plasticity induced by low level of BDNF could lead to lower responses to antidepressants and lower remission rates, resulting in delayed recovery from depression. This could be demonstrated by the results that “Responders” to treatment (≥50% improvement in depression ratings) had higher pretreatment BDNF levels than did “Nonresponders” [31]. Accordingly, the BDNF gene appears to be a potential candidate mediator of mechanisms relevant to the pathogenesis of depression. In the present study we also discovered reduction of BDNF expression in the hippocampus of RRS model and the elevation after Shuyu treatment. Furthermore, the increase of BDNF induced by Shuyu is mediated by MOR, as it was strengthened by CTAP, a selective MOR antagonist. Combined with the results that basal expression of BDNF in MOR-KO mice hippocampi was significantly lower than in wild-type mice [24], our data suggest the critical role of MOR in the behavioral sequelae of psychosocial stress and consequent regulation of BDNF expression.

We further investigate the link between MOR inhibition and BDNF increase induced by Shuyu. CREB, one of the members of nuclear factors family, may bind to the transcription element of numerous genes and regulate the transcription, such as BDNF. CREB have been implicated in the etiology and development and treatment of depression. CREB protein expression in the brain region was decreased in the depression models and reversed by antidepressants [32]. In depressed patients, T-lymphocytic CREB biomarkers are reduced and may assist in the prediction of response to SSRI drugs in depression [33]. Here we still found reduction of CREB phosphorylation in RRS-induced depression rats. That is to say, depression-like behaviors in our study were accompanied by inactivation of CREB, while the Shuyu administration restored the activity of CREB. Importantly, this restoration of Shuyu on CREB activity is associated with MOR, as the combination use of Shuyu and MOR antagonist CTAP further increased the CREB activity.

Moreover, the pathway of shc/Frs2 and ras/raf-mediated activation of a MAPK phosphorylation cascade involving MEK and ERK1/2 is the most important signal pathway following the binding of BDNF and its receptor TrkB. Recent studies suggest that MEK and ERK1/2 signaling participate in the neurobiological response to chronic stress and in the molecular activity of antidepressants. Acute elevated platform stress induced reduction of phosphor-Ser217/221-MEK in the rat prefrontal cortex, which could be reversed by treatment with antidepressants tianeptine and imipramine [34]. Similarly, in traumatic brain injury-induced depression rats, decrease in the phosphorylated levels of ERK1/2 could be detected and then reversed by fluoxetine, a popular antidepressant [35]. In consistent with these results, here we still showed inhibition of phosphorylation of MEK and ERK1/2 in RRS-induced rats. And these impairments could be rescued with Shuyu treatment in a MOR-dependent manner.

In fact, phosphorylation of ERK1/2 may initiate numerous gene transcriptions, of which CREB is the common one. CREB will further promote some gene transcription, including BDNF. This has been reported in some experimental studies. Takano et al. found that imipramine induced BDNF expression in astrocytes through CREB activation via MEK/ERK pathways [36]. Likewise, MEK, ERK1/2, and CREB signaling pathways are involved in PGE2-induced BDNF synthesis in rat dorsal root ganglion neurons [37]. Therefore, MEK, ERK1/2, CREB, and BDNF may compose a signal loop and play critical roles in the neural plasticity and etiology and treatment of depression. Activation of this signal loop may be seen from Shuyu treatment and subsequent MOR downregulation. Further complete and concise signal pathway should be explored in the future study.

## 5. Conclusion

Taken together, the present study further confirmed the antidepressant-like effects of Shuyu in the RRS exposed rats. Moreover, our results demonstrated that the antidepressant-like effect of Shuyu is closely associated with activation of hippocampal MEK-ERK-CREB-BDNF signal loop. Meanwhile, the above activation of Shuyu may at least in part be due to inhibition of MOR. MOR might be an important potential target in the development of antidepressants. Also, Shuyu is worthy of further investigation as a potential antidepressant.

## Figures and Tables

**Table 1:** 

Peaks	Constituents	Concen. (mg/g)	Linearity	LOD (*μ*g/L)	LOQ (*μ*g/L)	CV (%)
1	paeoniflorin	6.01 ± 0.1	*y* = 3.75 × 10^4^ + 5.27 × 10^2^ (6.74–67.4 *μ*g/mL) R = 0.9998	3.2	7.2	6.21

2	liquiritin	1.6 ± 0.03	*y* = 2.69 × 10^5^ − 1.39 × 10^2^ (4.84–48.4 *μ*g/mL) R = 0.9999	1.8	4.9	5.23

3	ammonium glycyrrhetate	6.9 ± 0.06	*y* = 6.85 × 10^4^ + 6.81 × 10^2^ (7.2–70.2 *μ*g/mL) R = 0.9999	7.6	13	7.11

4	saikoside a	3.6 ± 0.04	*y* = 1.38 × 10^4^ + 4.61 × 10^2^ (16.7–167 *μ*g/mL) R = 0.9999	6.5	12	6.87

5	saikoside d	1.68 ± 0.05	*y* = 7.12 × 10^4^ − 6.21 × 10^2^ (21.2–212 μg/mL) R = 0.9997	2.3	7	8.03

Data is expressed as mean ± standard deviation in mg/g of raw herbal materials calculated from three batches.

Inter-batch coefficients of variation (CV) were calculated across three batches of Shuyu preparation.

## Abbreviations

BDNF: Brain derived neurotrophic factor
CREB: cAMP response element binding protein
CTAP: D-Phe-Cys-Tyr-D-Trp-Arg-Thr-Pen-Thr-NH2
MOR: *μ* opioid receptor
RRS: Repeated restraint stress.
